# Peroral iron supplementation can be provided to piglets through a milk cup system with results comparable to parenteral iron administration

**DOI:** 10.1093/tas/txab004

**Published:** 2021-01-12

**Authors:** Nadia Jakobsen, Marie Louise M Pedersen, Charlotte Amdi

**Affiliations:** 1 Department of Veterinary and Animal Sciences, Faculty of Health and Medical Sciences, University of Copenhagen, Frederiksberg C, Denmark; 2 SEGES Danish Pig Research Centre, Copenhagen, Denmark

**Keywords:** growth performance, hemoglobin level, iron, milk replacer, pigs

## Abstract

The objective of this study was to investigate if iron can be allocated to piglets through sow milk replacer fed in a milk cup system with efficacies comparable to intramuscular (IM) administration of 200 mg gleptoferron. Two hundred and ninety-four piglets from 21 litters were allocated to three different iron treatments (*n* = 98). The treatments were 1) Control (CON) provided no supplemental iron, 2) Injected iron (II) provided 200 mg gleptoferron IM on day 3 postpartum, and 3) Milk iron (MI) provided sow milk replacer “DanMilk Supreme” added 1 % “Piglet Boozt” ad libitum from day 0 until 21 days postpartum. All piglets had access to dry feed from day 6. Initial and final body weight was registered and hemoglobin (Hb) levels were analyzed on day 0, 3, 7, 10, 17, and 21 after farrowing. In order to correlate drinking pattern with Hb level and growth, video cameras were installed, and drinking pattern was recorded on day 18 postpartum. A blood sample was drawn from piglets from three litters per treatment on day 21 for a complete hematology profile. The results showed that iron treatment had an effect on Hb levels (*P* < 0.001) that were different between all groups from day 10, resulting in a mean Hb level of 76.2 g/L (CON), 120.9 g/L (II), and 105.4 g/L (MI) on day 21. The mean Hb level for both MI and II was above the anemia threshold of 90 g/L and the Hb level of the II group was above 110 g/L and the piglets thus defined as normal. Treatment had a significant effect on Hb, hematocrit (hct), mean corposcular volume (MCV), mean corpuscular hemoglobin (MCH), red blood cell distribution width (RDW), lymphocytes (%), neutrophils (bill/L) (*P* < 0.05), neutrophils (%) (*P* < 0.01), with CON being significantly different from MI and II that were similar or tended to differ. Total visits at the cup was not correlated to Hb level (*r* = 0.08) and growth was not affected by treatment (*P* = 0.99). It is concluded that iron can be supplemented to piglets through a milk cup system with efficacies comparable to parenteral administration of 200 mg gleptoferron.

## INTRODUCTION

Pigs are born with limited iron reserves and due to a high growth rate, a restricted supply of iron from sow milk (Venn et al., 1947) and limited access to natural iron sources, pigs experience a physiological drop in hemoglobin (Hb) in the first days postpartum (Ventrella et al., 2017). If the piglets are not supplemented with iron, they will develop iron deficiency (Hb < 110 g/L) or iron deficiency anemia (IDA) (Hb < 90 g/L) (Egeli et al., 1998; Bhattarai and Nielsen, 2015; Stojanac et al., 2016). Pigs suffering from anemia will have reduced feed intake (Knight and Dilger, 2018) and body weight (Loh et al., 2001) both before and after weaning and increased mortality post weaning (Stojanac et al., 2016). In addition, anemic pigs will show clinical signs such as paleness, lethargy, and weakness (Smith, 1997). Securing a sufficient iron status in piglets is therefore of great importance for both the welfare and the performance of the piglet.

Currently, iron can be administered either perorally or parenterally, with intramuscular (IM) injection of iron between day 0 and day 4 postpartum being the most common method. Injection, increases the risk of infections, overdosing, causes stress to the animals and is time consuming. In addition, parenteral administration of iron shows conflicting results, with newer on farm screenings from Canada, France, Denmark, and United States, finding 6–25 % of piglets with IDA despite injections with 200 mg iron after farrowing (Perri et al., 2017; Brilland et al., 2019; Møller and Pedersen, 2019; Olsen, 2019). To avoid the detrimental effects of injections, oral preparations can be allocated either as paste, micro emulsion, as liquid iron in the drinking water, as oral iron fed on the floor or supplemented in the diet. However, the efficacy of these methods are equivocal (Sørensen, 1998; Loh et al., 2001; Maes et al., 2011), and several studies found a decreased efficacy compared to injections (Sørensen, 1998; Stojanac et al., 2016). Furthermore, oral preparations such as pastes and emulsions require individual, manual and several administrations to increase efficacy, which make labor similar or higher than parenteral administration.

An alternative to parenteral iron, which would decrease labor, problems associated with parenteral administration and allow the piglets to control their own iron uptake, is to provide iron automatically through a milk cup system and through ad libitum access to dry feed. However, milk- and dry-feed intake is associated with large within and between litter variation (Sørensen, 2017) and the intake is relatively low (Novotni-Dankó et al., 2015). Therefore, the aim of this study was to investigate if iron can be supplemented to piglets through ad libitum access to sow milk replacer added peroral iron and dry feed and to evaluate if this concept could provide Hb levels comparable to an IM injection of 200 mg gleptoferron.

## MATERIAL AND METHODS

### Animal Ethics Statement

The experiment was approved by the Danish Animal Experiments Inspectorate and the Danish Medical Agency (j.nr. 2018-15-0201-01515).

### Animals and Experimental Design

The experiment was conducted in a commercial pig herd with ~750 sows (Landrace × Yorkshire [LY]) mated with Duroc (Danbred P/S, Herlev, Denmark). Twenty-one sows (parity 1–7) and their 294 piglets were included in the trial and the piglets were allocated to one of three treatments, with 98 piglets in each group. The treatments were 1) Control (CON) provided no supplemental iron (negative control group), 2) injected iron (II) provided 1 mL of Viloferron containing 200 mg gleptoferron (iron4u, Holte, Denmark) IM in the neck on day 3 postpartum and 3) Milk iron (MI) provided sow milk replacer added 1 % “Piglet Boozt” (Agilia A/S, Videbæk, Denmark) ad libitum from day 0 until 21 days postpartum. In the MI group, 400 mL of “Piglet Boozt” was added automatically to 40 L of milk replacer in the milk system (Pump’N’Grow Fresh, Agilia A/S, Videbæk, Denmark) when a new portion of milk replacer was mixed. “Piglet Boozt” contained 26,000 mg FeSO_4_·H_2_O per kg corresponding to 11,440 mg Fe/40 L of milk, equivalent to a dose of 2,315.5 ppm FeSO_4_·H_2_O and vitamin B12, Zinc, organic acids, energy, and salts. All piglets had access to dry feed from day 6 postpartum.

The experiment commenced the morning after farrowing on the two major farrowing days in the herd (denoted day 0) and contained two batches. Before litter standardization, sows that had farrowed within the last 24 h and had given birth to more than 14 piglets, were assigned to one of the three test groups balanced by parity. All liveborn piglets were weighed (initial body weight) and the litter standardized by removing the smallest and largest piglets until the litter contained 14 of the sow’s own piglets. After standardization, no piglets were moved between or within groups. A colored ear tag was inserted in the piglet’s left ear for identification and baseline Hb measurements were performed on farm using HemoCue Hb 201+ (HemoCue, Brønshøj, Denmark). Thereafter, the milk was turned off for litters in-group CON and II and a milk replacer supplemented with iron was turned on for the litters in the MI group. Continuous Hb measurements were performed on day 0, 3, 7, 10, 17, and 21 using HemoCue and an additional blood sample was taken on day 21 from 3 litters per group. Cameras were installed above the pens of the MI group in order to record the drinking pattern of the piglets at day 18, to correlate drinking pattern with Hb levels and growth. If a pig, due to health or other problems had to be removed, the date and cause was noted, and the piglet was moved to a sow outside the trial. The day after farrowing, piglets had their teeth grinded and at 3–4 days of age the tails were docked, males were castrated, and piglets were treated with Baycox (Bayer Animal Health GmbH, Leverkusen, Germany). The piglets were cared for according to normal procedures on farm and the experiment was completed on day 21 were the final body weight was recorded.

### Housing, Feeding, and Management

Sows were inserted in the farrowing unit 3–4 days before expected farrowing. Pens measured 4.06 m^2^ and contained crates that were 2.00 m long and 0.60 m wide. The floor in the pens consisted of 1/3 slatted and 2/3 solid floor and there was a covered creep area in the corner of the pen. Straw was provided on the floor as nesting material until farrowing to stimulate nest building behavior and behind the sow at farrowing to limit heat loss after birth. The piglets were provided with extra heat until day 7 from a heating lamp in the covered creep area with floor heating. The temperature in the units was set at 22 °C for the entire lactation period. The units were ventilated using diffuse ventilation.

In the farrowing unit, the sows were fed individually with three feedings per day until farrowing. An additional feeding was added when all sows had farrowed. The sows received 2.4 kg/day of a diet consisting of barley, wheat, soybean meal, sugar beet pellets and soy oil as the main ingredients and a mineral and vitamin mixture containing 164.72 ppm Fe (energy content 12.6 ME/kg and 8.3 SID lysine/kg and 131 g SID CP/kg). In addition, the farm supplemented the standard diet with 1 kg of an energy rich meal (cake residuals). The feed intake was gradually increased to 10 kg/day during lactation. The sows and piglets were provided with water through drinking nipples ad libitum. The piglets were supplemented with dry feed on the floor from day 6 containing barley, wheat, dehulled soybean meal, HP 300 (Hamlet Protein, Horsens, Denmark), soy oil, DanStart 18 (Vilomix, Mørke, Denmark) and a mineral and vitamin mix with 187.54 ppm Fe (Nutrimin A/S, Ans By, Denmark). Additionally, the MI group had ad libitum access to sow milk replacer (DanMilk Supreme 1.0, Agilia A/S, Videbæk, Denmark) from litter standardization until weaning. The milk replacer consisted of dairy by-products, whey protein, sweetened whey protein and a mineral and vitamin mixture with 150 ppm Fe. The milk replacer was provided in small cups installed next to the drinking nipples, outside the reach of the sow. The milk system automatically mixed 40 L of milk every hour with 125 g milk powder per L water and the milk tank was cleaned daily according to the manufacturer’s instructions.

### Blood Analysis

The baseline Hb analysis (day 0) and the Hb analysis on day 3, 7, 10, 17, and 21 was performed on farm with HemoCue. Blood was drawn by puncture with a 23-gauge needle (Kruuse, Langeskov, Denmark) of the capillaries in the piglet’s ear and 10 μL blood was collected. Blood for a complete hematology analysis was collected on day 21 postpartum from three litters per group (CON: *n* = 37, II: *n* = 28, MI: *n* = 28). The blood was drawn in dorsal recumbency by jugular vein puncture with a 22-gauge needle (BD Vacutainer, Franklin Lakes, NJ) and dispensed into 6 mL vacutainer EDTA tubes (BD Vacutainer, Franklin Lakes, NJ). The tubes were kept cool and analyzed using the Advia 2120i Haematology System (Siemens Healthcare Diagnostics Inc, Tarrytown, NY), the following morning (Central Lab, Frederiksberg, Denmark).

### Video Recordings

Seven cameras (IPC-HDW1431S, Dahua technology, Hangzhou, China) were installed above the seven sows in the MI group and turned on, on day 18. The cameras were recording for 48 h to ensure enough footage to get 24 h of consecutive video recordings per sow. Prior to the recording all piglets in the litter were identified with a number from 1 to 14 written on their back with a black pen (Edding International GmbH, Ahrensburg, Germany). The written number was correlated to the ear tag of the pig so Hb levels and growth could be compared to drinking pattern. Recordings were made for 24 h starting at 00.00 and commencing at 23.59 on day 18 for four sows and from 11.00 on day 17 until 10.59 on day 18 for three sows. The video analysis was performed using MSH (MSH Svidia client, 6_0_12_428_i8_ufl) and a recording was made every time a piglet visited the milking cup (snout in cup). If a piglet removed its head fully from the cup and then continued drinking a new registration was made. In total, drinking patterns of 91 piglets were included in the analysis.

### Statistical Analysis

All statistical analyses were performed in the statistical program R (version 3.4.4), using the linear mixed effects model with piglet as the experimental unit. The primary test parameter was Hb levels and secondary test parameters were growth, final body weight, initial body weight and haematology. For all test parameters sow ID was included as a random effect and litter size, parity and treatment were included as fixed effects except for the analysis of the blood parameters where, treatment and batch were included as fixed effects. In the analysis of Hb levels, the interaction between treatment and day was included as a fixed effect, baseline Hb and initial weight as covariates and piglet ID was included as a repeated measure using the Gaussian serial correlation structure. For the analysis of growth, final body weight and the blood parameters, initial body weight was included as a covariate. If assumptions of normality and homogeneity of variance were not met, data was either transformed and/or outliers were removed. For all parameters model reduction was performed using ANOVA and systematic effects with a significance level *P* > 0.10 was excluded, except the effect of treatment and drinking pattern, which was maintained regardless of significance level since these were the primary factors of interest. Means were extracted with EMMEANS and results presented as least square means ± SEM. For the transformed data, means were presented as back transformed means. Results were considered significant at *P* < 0.05 and a tendency at *P* < 0.10 and all interactions between included factors were tested.

## RESULTS

The experiment included 294 piglets, from 2 batches, born from 21 sows, with 7 litters and 98 piglets allocated to each treatment (CON, II and MI). On day 0 after weighing, ear tagging and litter standardization but before baseline Hb level was analyzed, one piglet from the MI group died, hence baseline Hb levels for 293 piglets were included in the statistical analysis. Between day 0 and day 21, 15 piglets died; 4 in-group CON, 10 in II and 1 in the MI group and 3 piglets were moved to sows outside the trial. Furthermore, 10 piglets lost their ear tag and could not be identified. Blood was drawn from three litters per treatment on day 21 resulting in a complete hematology analysis from 103 pigs.

### Hb Levels

**Figure 1.**
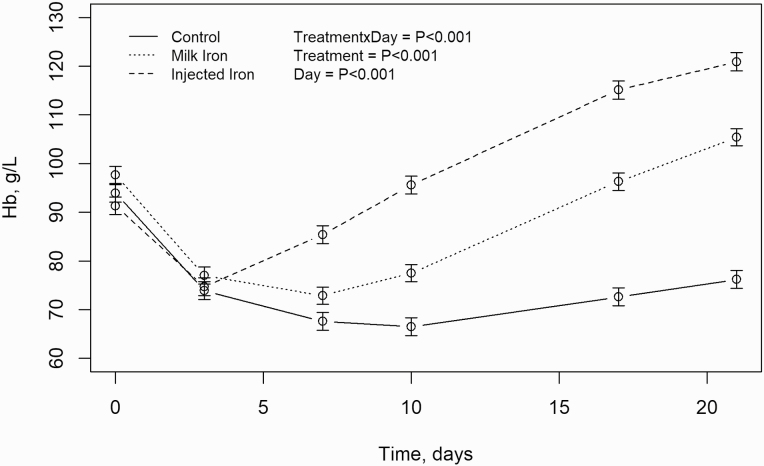
The average development in Hb levels from day 0 postpartum until day 21 for the control (CON) group, injected iron (II) group and the milk iron (MI) group – means ± SEM.

The development in Hb levels between the three groups is illustrated in Figure 1. After farrowing, the piglets experienced a drop in Hb levels to below the anemic threshold. Hereafter, the Hb levels increased in the II piglets between day 3 and 7, whereas the Hb levels continued to decline until day 7 in the MI group and day 10 for the CON group (Figure 1). The results from the data analysis can be found in Table 1. An interaction was found between treatment and day (*P* < 0.001) and there was a significant effect of treatment (*P* < 0.001), initial body weight (*P* < 0.001), baseline Hb level (*P* < 0.001), day (*P* < 0.001) and litter size (*P* = 0.015). The baseline Hb levels (day 0) did not differ significantly between treatments (*P* > 0.05), but there was a tendency toward a higher baseline level in MI compared to II (*P* = 0.068). No significant difference was seen between treatments on day 3 (*P* > 0.05). On day 21, II had a higher mean Hb level than MI (*P* < 0.01) and CON (*P* < 0.001) and CON was significantly lower than MI (*P* < 0.001). The mean Hb level for both MI and II was above the anaemia threshold of 90 g/L and the Hb level of the II group was above 110 g/L and the piglets thus defined as normal (Bhattarai and Nielsen, 2015). In Table 2, the percentage of piglets categorized as being anaemic, iron deficient or normal on day 21 and the average iron uptake can be found. In the control group 70 % of the piglets were defined as anemic compared to 4.8 and 15.4 % for the II and MI group, respectively. Furthermore, only 4.4 % of the CON piglets had normal Hb levels compared to 83.3 and 48.8 % in the II and the MI group. The number of piglets with Hb levels above 90 g/L was lowest in the control group. When calculating the average iron uptake based on the difference in Hb levels from day 0 to 21 postpartum, it can be seen that both the MI and II group have an iron uptake above the 200 mg Fe, where as the average iron uptake is far below 200 mg in the Con group (131.5 mg Fe). However, the variation in Hb levels is high throughout the entire experimental period, causing a large variation in Hb levels at day 21 that are not affected by treatment. The minimum and maximum values in the C group were 33.0 and 112.0 g/L, in the II group they were 50.0 and 149.0 g/L and for the MI group 69.0 and 169 g/L. Furthermore, a large within litter variation was seen in baseline Hb levels, that could not be explained by initial body weight. The variation in Hb levels can be seen in Supplementary Figure 1.

**Table 1.** Mean Hb levels of piglets not supplemented iron (CON), provided parenteral iron (II) and provided peroral iron fed in a milk cup system (MI) from day 0 to day 21 postpartum

					*P*		
	CON	II	MI	SEM	Treatment	Day	Treatment × day
*n*	90–98	84–97	91–98				
Day 0	93.9	91.3^†^	97.7^†^	1.75	*P* < 0.001	*P* < 0.001	*P* < 0.001
Day 3	73.9	74.7	77.0	1.75			
Day 7	67.6^b^	85.4^a^	72.9^b^	1.75			
Day 10	66.5^c^	95.6^a^	77.5^b^	1.75			
Day 17	72.6^c^	115.1^a^	96.3^b^	1.78			
Day 21	76.2^c^	120.9^a^	105.4^b^	1.78			

Data show the mean Hb levels measured for each of the three treatments. Values within a row with a symbol (†) shows a tendency toward a difference (*P* < 0.10).

^a,b,c^ Values within a row with different superscripts differ significantly (*P* < 0.05).

**Table 2.** The distribution of normal, iron deficient and anemic pigs at day 21 postpartum and average mg Fe uptake for piglets provided no supplemental iron (CON), provided parenteral iron (II) and provided peroral iron fed in a milk cup system (MI)^*^

	CON	II	MI
*n*	90	84	91
Anemic	70 % (63)	4.8 % (4)	15.4 % (14)
Iron deficient	25.6 % (23)	11.9 % (10)	36.3 % (33)
Normal	4.4 % (4)	83.3 % (70)	48.4 % (44)
Avg. iron uptake, mg^†^	131.5	261.0	242.6

Values expressed as percentage (amount).

^*^Anemic: Hb < 90 g/L, iron deficient: 90 ≥ Hb < 110 g/L, normal: Hb ≥ 110 g/L (Bhattarai and Nielsen, 2015).

^†^Average iron uptake was calculated based on the increase in Hb levels from day 0 to 21 postpartum.

### Growth Performance

The growth performance was not affected by treatment (*P* > 0.05), however, initial body weight and parity had a significant effect on growth (initial body weight–final body weight) (*P* < 0.001). The mean initial body weight, final weight and growth can be seen in Table 3. No correlation was found between growth and Hb levels (*r* = 0.102) or initial weight and baseline Hb levels (*r* = −0.034) and supplementing the piglets with sow milk replacer did not increase growth significantly in the MI group compared to the CON and II groups.

**Table 3.** The growth performance of piglets provided no iron (CON), parenteral iron (II) or iron through milk replacer (MI)

	CON	II	MI	SEM	*P*
*n*	98	96	98		
Initial body weight, kg	1.19	1.39	1.28	0.145	0.63
Final body weight, kg^*^	6.02	6.13	6.10	0.755	0.89
Growth, kg^*^	4.65	4.76	4.74	0.755	0.99

Data presented as least square means.

^*^ *n*= 90, 83, and 91 for the CON, II and MI group, respectively.

### Drinking Pattern

**Figure 2.**
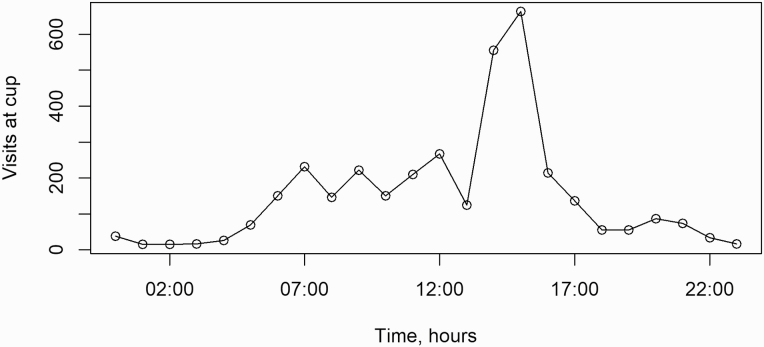
An illustration of the diurnal pattern of piglets from the MI group in visits at the cup on day 18 postpartum.

**Figure 3.**
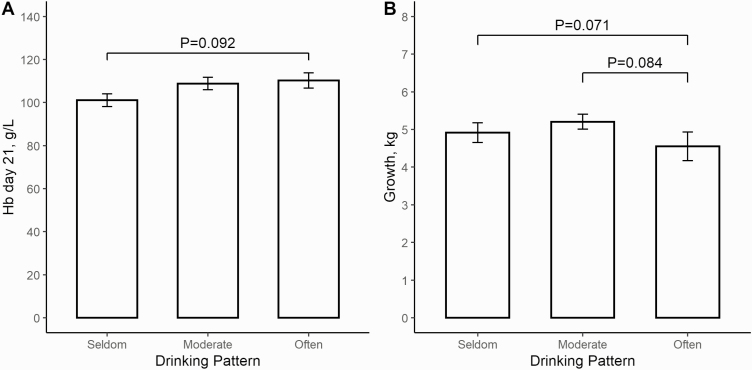
(A) Barplot with SEM and significance levels of the Hb level on day 21 for piglets from the MI group drinking seldom (<23 visits in 24 h), moderate (between 23 and 48 visits in 24 h) or often (>48 visits in 24 h). (B) Barplot with SEM and significance levels of growth for piglets drinking seldom, moderate, or often.

In the MI group, piglets on average visited the cup 39 times during the 24 h registered and all piglets visited the cup at least once with 92 % visiting the cup more than 10 times per 24 h. Visits at the cup varied from 1 to 123 visits per piglet and followed a diurnal pattern. As seen in Figure 2, the piglets visited the cup more frequently during the day with a large peak between 13.00 and 16.59. When classifying the drinking pattern as piglets drinking seldom (<23 visits in 24 h), moderate (between 23 and 48 visits in 24 h) and often (>48 visits in 24 h) a tendency (*P* = 0.092) toward lower Hb levels on day 21 for piglets drinking seldom was found compared to piglets drinking often. The effect of drinking pattern on mean Hb levels and growth can be seen in Figure 3A and B. No significant difference was found between piglets drinking seldom and moderate (*P* > 0.05) or moderate and often (*P* > 0.05). When comparing drinking pattern and growth, a tendency toward a higher growth was seen in piglets drinking moderate compared to often (*P* = 0.084) and seldom compared to often *(P* = 0.071), but no difference was seen between moderate and seldom (*P* > 0.05). No correlation was found between total visits at the cup and Hb levels on day 21 (*r* = 0.08).

### Hematology Analysis

The hematology results can be found in Table 4. The piglets originated from two batches and a significant effect of batch was observed in the statistical analysis of mean corpuscular hemoglobin (MCH), basophils (%), and eosinophils (%) (*P* < 0.05) and a tendency was found in the analysis of basophils (bill/L) (*P* = 0.081) and mean corpuscular volume (MCV) (*P* = 0.055). Treatment had a significant effect on Hb, Haematocrit (Hct), MCV, MCH, red blood cell distribution width (RDW), lymphocytes (%), neutrophils (bill/L) (*P* < 0.05), neutrophils (%) (*P* < 0.01) and tended to have an effect on eosinophils (bill/L) (*P* = 0.058). Hemoglobin levels were affected by treatment, with CON having significantly lower Hb levels than II (*P* < 0.05) and tended to have lower Hb levels than the MI group (*P* = 0.069). No difference was seen between II and MI (*P* > 0.05). The Hct value tended to be lower in the CON group compared to both II (*P* = 0.051) and MI (*P* = 0.098) but no difference was observed between MI and II (*P* > 0.05). For MCV and MCH, CON piglets had lower values compared to II (*P* < 0.01) and MI (*P* < 0.05) and MI showed a tendency toward lower values than II (MCV: *P* = 0.066, MCH: *P* = 0.091). For the RDW, percentage of neutrophils and lymphocytes and the total amount of neutrophils the C group differed significantly from the II (*P* < 0.05) and MI (*P* < 0.05) and no difference was seen between II an MI (*P* > 0.05). The total amount of eosinophils tended (*P* = 0.086) to be higher in the CON group compared to II.

**Table 4.** The hematology results of piglets provided no iron (CON), parenteral iron (II) or iron through milk replacer (MI)

	CON	II	MI	SEM	*P*
*n*	37	38	28		
Total leukocytes, bill/L	12.9	9.7	9.9	0.96	0.097
Total erythrocytes, bill/L	5.4	5.5	5.8	0.23	0.590
Hemoglobin, mmol/L	5.23^b†^	6.81^a^	6.53^a†^	0.30	**0.023**
Hematocrit, L/L	0.29^†^	0.36^a†^	0.35^a†^	0.015	**0.034**
MCV, fL	55.4^a^	66.0^†^	61.1^†^	0.98	**0.002**
MCH, fmol	0.99^a^	1.24^†^	1.14^†^	0.022	**0.001**
MCHC, mmol/L	17.7	18.7	18.5	0.03	0.116
RDW, pct	24.3^a^	18.6^b^	19.5^b^	0.97	**0.013**
Thrombocytes, g/L	472	333	365	40.3	0.120
MPV, fL^1^	13.1	12.7	11.6	1.05	0.634
Neutrophils, pct	51.3^a^	40.0^b^	40.4^b^	1.84	**0.012**
Lymphocytes, pct	36.9^b^	48.7^a^	48.3^a^	2.32	**0.028**
Monocytes, pct ^1^	3.8	3.3	3.5	0.39	0.715
Eosinophils, pct	4.7	4.4	4.8	0.54	0.955
LUC, pct	1.4	1.3	1.4	0.25	0.956
Basophils, pct ^1^	0.27	0.33	0.25	0.021	0.239
Neutrophils, bill/L	5.71^a^	3.82^b^	4.16^b^	0.245	**0.008**
Lymphocytes, bill/L^1^	4.32	4.43	4.44	0.495	0.983
Monocytes, bill/L^1^	0.47	0.35	0.36	0.072	0.515
Eosinophils, bill/L^1^	0.60^†^	0.39^†^	0.44	0.043	0.058
LUC, bill/L^1^	0.16	0.10	0.12	0.026	0.754
Basophils, bill/L	0.04	0.03	0.03	0.003	0.322
Reticulocytes, bill/L^2^	394	481	479	75	0.684
Reticulocytes, pct^2^	7.55	8.38	8.41	1.26	0.867

LUC = large unstained cells; MCHC = mean corposcular hemoglobin concentration; MPV = mean platelet volume.

^1^The least square means have been back transformed (logarithmic transformation). Values within a row with different superscripts differ significantly (*P* < 0.05).

^2^ *n* = 26, 20, and 24 for CON, II and MI, respectively.

^†^Values within a row with a symbol (†) shows a tendency toward a difference (*P* < 0.10).

### The Agreement Between the HemoCue^®^ Hb 201+ and Advia 2120i Analysis

At day 21, postpartum Hb measurements were performed using the HemoCue^®^ Hb 201+. In addition, blood was drawn from three litters per treatment and a complete hematology analysis was performed (Advia 2120i). The Hb levels from the two analyses showed a correlation coefficient of 0.965 and the correlation can be seen in Supplementary Figure 2A. Furthermore, the agreement between the two methods was determined using the Bland-Altman analysis illustrated in Supplementary Figure 2B. The limits of agreement were −11.669 to 13.445 with a bias of 0.888 corresponding to the dashed and solid lines, respectively.

## DISCUSSION

Hemoglobin levels were associated with treatment and from day 10 all treatments differed significantly. In line with expectations and findings made by other studies, the Hb levels in all groups decreased shortly after birth, due to the expanding blood volume, caused by rapid growth (Furugouri, 1975; Ventrella et al., 2017). Hereafter, Hb levels continued to decrease in the CON group until around day 10 where after a small increase in Hb levels was seen. Since the iron content in sow milk is generally low (Venn et al., 1947) and iron from external sources such as sow feed and sow feces are considered limited in pens with slatted floor and raised sow creeps, the increase might be associated with dry feed intake. Dry feed was available from day 6 and studies suggest a fast utilization, with peroral iron being incorporated into the erythrocytes within a couple of days after ingestion (Furugouri, 1975). Hence, the rise in Hb levels in the CON group indicates that providing piglets with dry feed from an early age did encourage dry feed intake. However, the intake was not sufficient at restoring iron status and the mean Hb level in-group CON was 76.2 g/L at day 21 postpartum. A value comparable to Hb levels found in other studies (Svoboda et al., 2007; Ettle et al., 2008; Stojanac et al., 2016). In addition, 70 % of the CON piglets had Hb levels below 90 g/L and were thus defined as anemic and only 4.4 % was defined as normal at day 21 (Hb > 110 g/L). Injecting iron at day 3 postpartum increased Hb levels rapidly and a mean Hb level of 120.9 g/L was reached, whereas MI increased the Hb levels more slowly to a total of 105.4 g/L. This difference in efficacy could apart from a difference in allocation method also be a result of different iron types, since the availability of iron is highly variable (Henry and Miller, 1995), but no studies comparing availability or utilization of peroral ferrous sulfate and parenteral gleptoferron were found. A hemoglobin level of 105.4 g/L is close to the “normal” limit of 110 g/L, however, the individual Hb measurements revealed that approximately 10—and 25 % more piglets were anemic and iron deficient, respectively, than when injections were used, leading to 4.8 and 15.4 % of II and MI pigs being anemic. This stresses the importance of stipulating the amount of pigs defined as anemic, normal or iron deficient and not just the mean Hb levels when comparing treatments since this might reveal the complexity of litter variation. The current study was an on farm study, which meant that the CON and II group, could not be provided with milk replacer without iron. This setup means, that it is impossible to distinguish the effect of other molecular differences in the diet, despite iron type and content, and if these differences could account for the increase in hemoglobin levels and the comparable hematology parameters between II and MI. However, the average iron intake calculated from increase in hemoglobin level between day 0 and 21, is comparable between the MI and II group and the mean Hb levels in the MI group is similar to Hb levels found by studies supplying 200 mg iron parenterally (Loh et al., 2001; Antileo et al., 2016; Stojanac et al., 2016).

The within litter variability in baseline Hb levels were high but the difference in baseline Hb levels could not be explained by initial body weight. According to a recent review on iron supplementation in suckling piglets, the placental iron transfer in pigs is poorly understood (Szudzik et al., 2018) and studies correlating Hb level at birth with birth weight has not been found. Venn et al., (1947), for example, compared birth weight and tissue iron from 10 piglets with inconclusive results. In the current study, litters were standardized before baseline Hb levels were measured resulting in a lower variation in birth weight in the litters. It can be speculated if a relationship between Hb levels and birth weight could be detected if Hb levels were measured before standardization. Another factor influencing Hb level at birth could be foetal hypoxia. Iron regulation is controlled by hepcidin synthesis in the liver, and it is affected by several factors such as, iron availability, inflammation, and hypoxia (Crichton, 2016). Hypoxia is a condition where the oxygen flow is compromised leading to a decrease in hepcidin synthesis and consequently a higher flow of iron to the circulation. Additionally, literature showed that sow hemoglobin levels have decreased (Bhattarai, 2019) and litter size increased (Hansen, 2019) during the last years and consequently the sow might not be capable of covering the iron needs of the piglets. However, even though litter size had a significant effect on Hb levels in this trial, no linear relationship could be detected between litter size and baseline Hb level. More research should be performed in order to explain the cause of the high variation in baseline Hb levels. A high variation in Hb responses was observed in all groups and the accuracy of the HemoCue^®^ Hb 201+ could perhaps account for the variability. A Bland-Altman analysis determined the limits of agreement between to be −11.669 to 13.445 with a bias of 0.888. For human use, the limits of agreement should be within ±5 g/L in order to classify HemoCue as precise enough to be used as an indicator of blood transfusion. However, the implications of a less accurate Hb result when HemoCue is used as a diagnostic tool in a pig herd is less severe and wider limits of agreement is assumed satisfactory. The CV for Advia2120i and HemoCue^®^ Hb 201+ was 19.89 and 23.07 %, respectively and together with the limits of agreement and the correlation the agreement is considered moderate and some variation in the result thus expected. In addition, the data suggests that HemoCue^®^ Hb 201+ tends to overestimate when Hb levels are below 80 g/L and underestimate when Hb levels are above 110 g/L.

Despite an anemia occurrence of 70 % in the CON group, no significant effect on growth or final body weight was detected between treatments in the current study. In contrast, several studies have found a decreased growth rate (Egeli et al., 1998; Loh et al., 2001; Stojanac et al., 2016) associated with a decrease in feed intake (Ettle et al., 2008) when pigs suffered from anemia. On average, the growth was 4.62 kg from day 0 to 21 and the end weight was 5.99 kg at day 21 in the CON group, which is comparable to bodyweights for piglets supplied with parenteral iron (Loh et al., 2001; Middelkoop et al., 2019). In addition, a maximum growth of 7.4, 9.0, and 7.8 kg for pigs in the CON, II, and MI group, respectively, was found, indicating that the growth rate was not compromised by any of the treatments. An explanation for the lack of effect on growth, could be that the piglets in group CON, were not anemic. However, Hb levels were on average 76.2 g/L on day 21 and the hematology analysis revealed significantly lower Hb, MCV, and MCH values and a tendency toward a lower Hct in the CON group compared to II and MI pigs. Furthermore, RDW was significantly higher in the CON group compared to both II and MI. The MCV is a measure of the size of the red blood cells (RBC), whereas MCH is a measure of the hemoglobin concentration in the RBC and RDW a measure of the variance in RBC volume. All three values, although significantly different from II and MI, fell within the reference interval determined by Perri et al., (2017) on 21-day-old piglets. The RBC in the CON group were significantly smaller and have a significantly lower Hb concentration than II and MI but are not classified as microcytic or hypochromic (Perri et al., 2017). In contrast, the Hb level in the blood and the Hct in the CON group fell outside the reference interval and several of the white blood cell indices were affected as well. Surprisingly, growth performance was unaltered, but the erythrocyte values indicated that CON piglets were negatively affected by the treatment and it can be speculated if an earlier blood test would show a microcytic and hypochromic anemia since Hb levels at day 21 were increasing.

Studies have shown that iron deficiency in pigs causes decreased neutrophil (Egeli et al., 1998; Svoboda et al., 2004) and leucocyte count (Svoboda et al., 2004), which is opposite to the findings in the current study. However, CON pigs had a significantly lower percentage of lymphocytes, compared to MI and II pigs, a finding supported by Svoboda et al. (2004). Low amounts of lymphocytes suggest that the pigs have not yet developed the adaptive immune response. Lymphocytes are an important part of the adaptive immune response and are responsible for recognizing antigens and activating the innate immune response, thus enhancing the efficiency of the immune system (Zimmerman et al., 2019). A decrease in lymphocytes would make the pigs more susceptible to opportunistic infections such as *E. coli* diarrhea (Daykin et al., 1982), which might explain why early weaned piglets, provided a low-iron diet to provoke IDA, suffered from a higher incidence of diarrhea compared to non-anemic piglets (Larkin and Hannan, 1985).

The piglets in the MI group all visited the milk cup at least once, with 92 % of the piglets visiting the cup more than 10 times in 24 h. This is a larger proportion than found by other studies on the subject, where 50–87 % of the piglets used the cup (de Greeff et al., 2016; Sørensen, 2017; Thorsen and Pedersen, 2019). It can be speculated if design and placement of the cup as well as flavor can influence visits at the milk cup, since design and flavor have been shown to affect creep feed intake (Middelkoop et al., 2019). In this study, the milk cup was a steel water drinker located next to the drinking nipple posterior to the sow and the milk replacer had a sweet apple taste due to sweetened whey in “Danmilk Supreme” and artificial apple flavor in “Piglet Boozt”. de Greeff et al., (2016), supplied the piglets with milk replacer manually twice daily in a round bowl on the floor, whereas, Sørensen, (2017) and Thorsen and Pedersen, (2019) used automatic round milking cups placed on the slatted floor and none of the milk replacers contained artificial flavor. More research into flavor, design, and placement and its effect on milk intake and usage of the cup should be performed to confirm this explanation.

The drinking pattern of the piglets followed a diurnal pattern comparable to findings by Sørensen, (2017) and Baumann et al., (2012), with a large peak during the afternoon and smaller peaks during the morning. Baumann et al., (2012) observed a large peak in the morning that was not apparent in the current study or in the study by Sørensen (2017). This could be due to disturbances caused by employee activity in the stable during the morning. From the video recordings, employees working in the stable seemed to disturb both piglet and sow activity, leading to less frequent nursings and more turmoil in the pens, but this was not systematically investigated. Literature on nursing interval and duration in sows have hypothesized that external disturbances could increase the level of incomplete nursings (Fraser, 1977), however, no studies investigating this directly was found. The many visits might be a consequence of more piglets drinking and piglets playing with and investigating the cup. In the trial, visits at the cup was used as an indicator of drinking behavior and it was hypothesized that visits at the cup day 18 would correlate with Hb levels at day 21. Visits at the cup was used as an indicator of milk intake, since individual milk intake cannot be established directly on farm and this measure has been used previously (Thorsen and Pedersen, 2019). However, total visits at the cup were not correlated to Hb levels, suggesting, that visits at the cup, might not be as indicative of milk intake as expected. As described above, growth was not influenced by any of the treatments, meaning that the access to supplemental milk did not enhance performance. This is in contrast to other studies, where access to sow milk replacer resulted in improved growth (Novotni-Dankó et al., 2015; Pedersen et al., 2019). Perhaps explained by a lower mixing ratio than recommended (125 g/L as opposed to 130–140 g/L). Additionally, all litters were standardized to 14 piglets, and no piglets were added to the litters if piglets died or were moved, thus the milk yield of the sows might have been large enough to cover the needs of the piglets. The piglets categorized as drinking often tended to grow less than piglets drinking seldom and moderate. In a study on drinking pattern in a Danish herd, piglets drinking often mainly fed on supplemental milk, whereas piglets drinking moderate or often gained nutrition from both the sow and the milking cup or mainly from the sow (Thorsen and Pedersen, 2019). Sow milk replacer contain less fat and less energy than sow milk (Theil et al., 2007) and piglets mainly consuming sow milk replacer have been shown to grow less efficiently than piglets nursing regularly from the sow (Thorsen and Pedersen, 2019), explaining why piglets that drank often grew less than piglets drinking seldom and moderate.

## CONCLUSIONS

In conclusion, providing piglets with peroral iron through a milk cup system increased hemoglobin levels to a mean value above the anemia limit (Hb > 90 g/L) and provided comparable growth rates to piglets injected with iron. In addition, the hematology analysis showed comparable hematology results between II and MI that were within the reference intervals. Providing piglets with dry feed but no supplementary iron did not modify growth, but severely increased the proportion of anemic piglets (Hb < 90 g/L) and significantly altered MCV, MCH, RDW related to iron status and the percentage of neutrophils and lymphocytes as well as the total amount of neutrophils related to immune function. Providing supplemental iron is crucial to maintain iron status and iron added to sow milk replacer and fed ad libitum in a milk cup system do provide hemoglobin levels comparable to parenteral iron supplementation and can, therefore, be used as a non-invasive supplement method in the farrowing unit.

## Supplementary Material

txab004_suppl_Supplementary_Figure_1

txab004_suppl_Supplementary_Figure_2

txab004_suppl_Supplementary_Figure_Legends
