# Supplementary figures and images for: Peroral iron supplementation can be provided to piglets through a milk cup system with results comparable to parenteral iron administration
Source: Transl Anim Sci. 2021 Jan 12;5(1):txab004. doi: 10.1093/tas/txab004 (PMC7881258; doi:10.1093/tas/txab004)

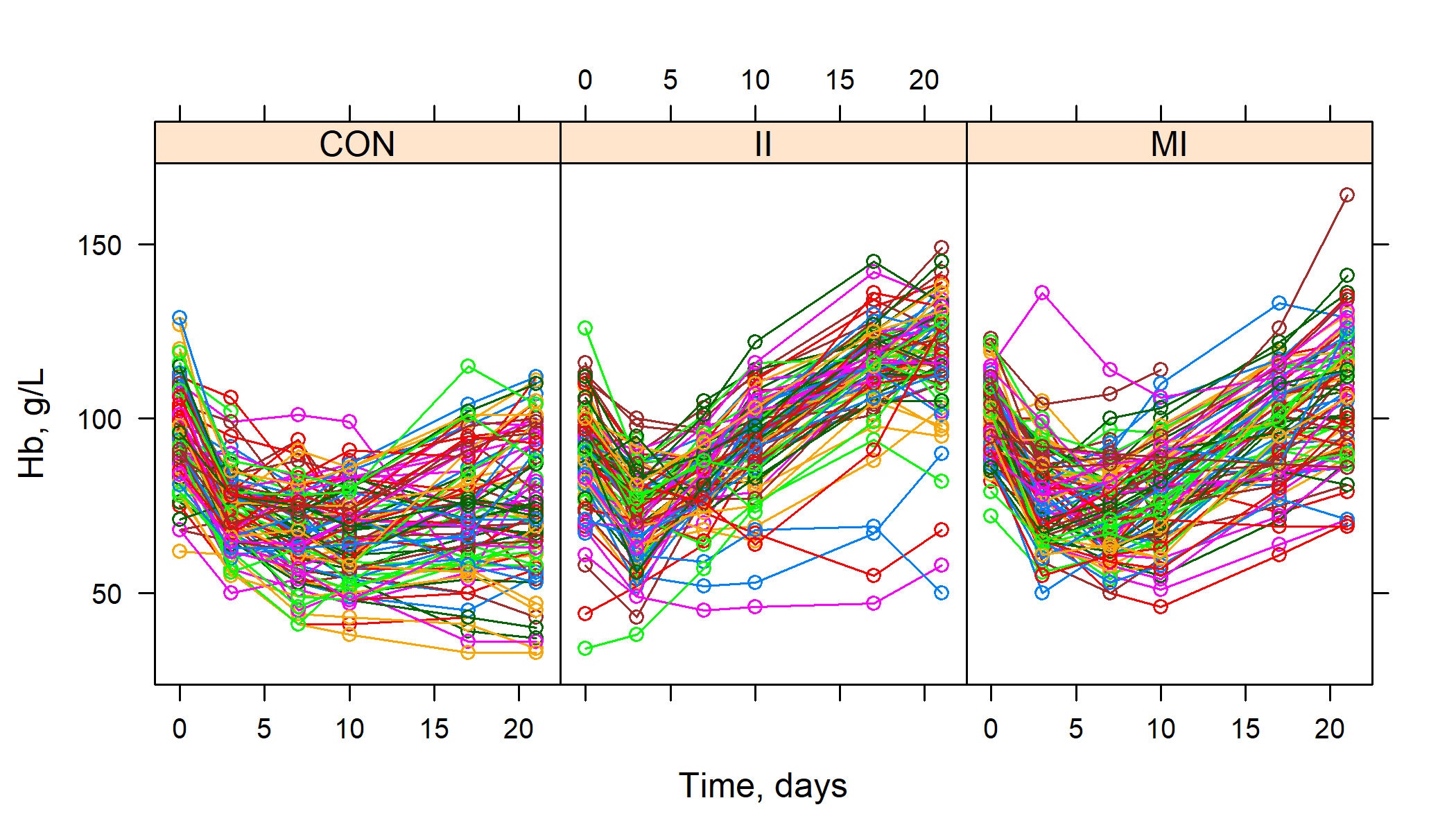

Supplement: txab004_suppl_Supplementary_Figure_1 [file txab004_suppl_supplementary_figure_1.png]

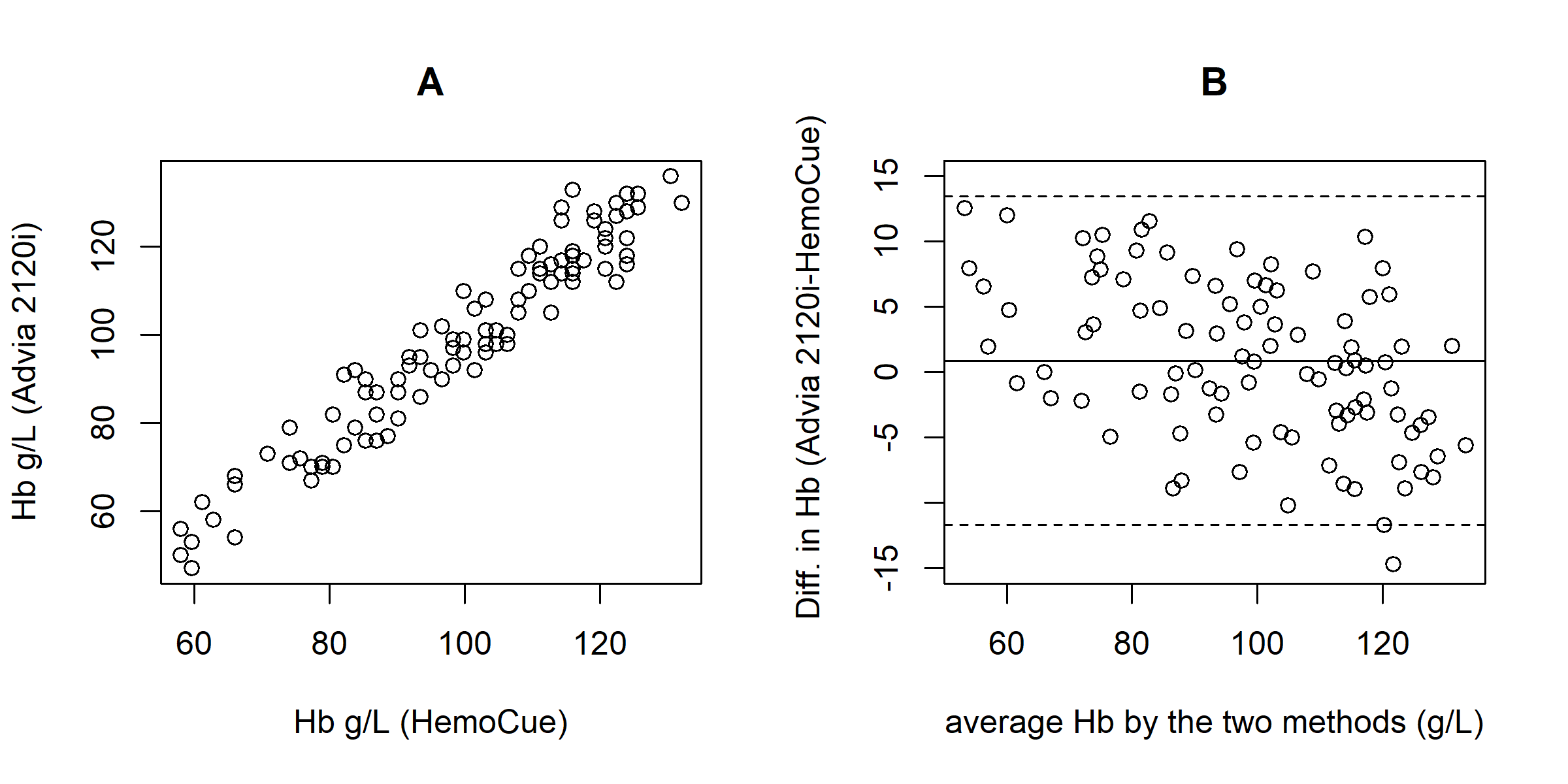

Supplement: txab004_suppl_Supplementary_Figure_2 [file txab004_suppl_supplementary_figure_2.png]
