# Supplementary material for: Peroral iron supplementation can be provided to piglets through a milk cup system with results comparable to parenteral iron administration
Source: Transl Anim Sci. 2021 Jan 12;5(1):txab004. doi: 10.1093/tas/txab004 (PMC7881258; doi:10.1093/tas/txab004)
Supplement: txab004_suppl_Supplementary_Figure_Legends [file txab004_suppl_supplementary_figure_legends.docx]

Peroral iron supplementation can be provided to piglets through a milk cup system with results comparable to parenteral iron administration^[[1]](#footnote-1)^.

Nadia Jakobsen^*^, Marie Louise M. Pedersen^†^ and Charlotte Amdi^*‡^

^*^Department of Veterinary and Animal Sciences, Faculty of Health and Medical Sciences, University of Copenhagen, 1870 Frederiksberg C, Denmark

^†^SEGES Danish Pig Research Centre, Axeltorv 3, 1609 Copenhagen, Denmark

^‡^Corresponding author: Charlotte Amdi. Email: ca@sund.ku.dk

Running title: Oral iron to piglets fed in a milk cup system

**Supplementary figure 1.** Subject profiles of the development in Hb levels from day 0 postpartum until day 21 for all piglets in the control group (CON), injected iron group (II) and milk iron group (MI)

**Supplementary figure 2.** A: An x-y plot of the haemoglobin levels provided by the HemoCue 201+ and by Advia 2120i. B: A Bland-Altman plot (Bland and Altman, 1986) of the difference in Hb (Advia 2120i-HemoCue 201+) and the average Hb level. The plot visualises the limits of agreement (The two dashed lines) calculated as d±2s. d = mean diff. and s = SD of the diff. The solid line corresponds to the mean difference. The Hb levels were with both methods and for both plots measured on day 21.

**References**

Bland, J. M., and D. G. Altman. 1986. Statistical methods for assesing agreement between two methods of clinical measurement. Lancet. 302–310.

1. [↑](#footnote-ref-1)
